# A Novel Transcription Factor VPA0041 Was Identified to Regulate the Swarming Motility in *Vibrio parahaemolyticus*

**DOI:** 10.3390/pathogens11040453

**Published:** 2022-04-10

**Authors:** Mingzhu Li, Hongmei Meng, Yang Li, Dan Gu

**Affiliations:** 1 Jiangsu Co-Innovation Center for Prevention and Control of Important Animal Infectious Diseases and Zoonoses, Yangzhou University, Yangzhou 225009, China; MZ120191191@yzu.edu.cn (M.L.); MX120170784@yzu.edu.cn (H.M.); MZ120171036@yzu.edu.cn (Y.L.); 2 Jiangsu Key Laboratory of Zoonosis, Yangzhou University, Yangzhou 225009, China; 3 Key Laboratory of Prevention and Control of Biological Hazard Factors (Animal Origin) for Agri-Food Safety and Quality, Ministry of Agriculture of China, Yangzhou University, Yangzhou 225009, China

**Keywords:** *Vibrio parahaemolyticus*, VPA0041, swarming motility, lateral flagellar system, RNA-seq

## Abstract

*Vibrio parahaemolyticus* can change their usual lifestyle of surviving in an aqueous environment attached to a host, wherein both swimming motility and swarming motility play important roles in lifestyle changes, respectively. VPA0041 is a novel transcription factor involved in regulating the swarming ability of *V. parahaemolyticus*. The deletion of the *vpa0041* gene resulted in the loss of swarming motility in the brain heart infusion (BHI) agars, while the swimming motility was unaffected by VPA0041. Transmission electron microscope (TEM) assays showed that no flagellum was found around the bacterial cells. RNA-sequencing (RNA-Seq) analysis revealed that VPA0041 regulated 315 genes; 207 genes were up-regulated, and 108 genes were down-regulated. RNA-seq results indicated that the lateral flagellar genes were down-regulated by VPA0041, which was confirmed by real-time quantitative reverse transcription-polymerase chain reaction (qRT-PCR). Electrophoretic mobility shift assays (EMSA) demonstrated that VPA0041 directly bound to the promoters of *vpa0264*, *vpa1548,* and *vpa1550* to regulate the expression of the lateral flagellar genes. Our results demonstrated that the transcription factor VPA0041 could directly regulate the expression of lateral flagellar genes to mediate the swarming motility in *V. parahaemolyticus*.

## 1. Introduction

*Vibrio parahaemolyticus* is a gram-negative bacterium found in various aqueous environments, including marine and coastal environments [1,2,3,4]. *V. parahaemolyticus* expresses two flagellar systems responsible for swimming motility and swarming motility, respectively [5]. Individual swimming behavior allows the bacteria to swim in an aqueous environment, while swarming is the movement of bacteria over solid surfaces or during host colonization [6]. Changes of environmental conditions are closely related to the disease outbreaks caused by bacterial pathogens. *V. parahaemolyticus* is a foodborne pathogen that can be influenced by changes of environment factors [7]. Understanding the factors that could influence the dissemination of pathogens in the environment is essential for controlling opportunistic pathogens.

The marine and freshwater environment is a valuable source for bacteria worldwide. *V. parahaemolyticus* has an intricate life cycle that depends on environmental conditions, and contains swimmer and swarmer cells for living in liquid and on solid environments, respectively [7]. The single polar flagellum is encoded by genes responsible for the swimming motility in liquid environments. When *V. parahaemolyticus* attaches to solid surfaces, the lateral flagellum is stimulated and responsible for the swarming ability [8,9,10,11,12]. A previous study showed swarm colonies could be a continuous source of cells released into the environment. The released cells could be spread in the liquid environment and attach to new surfaces, but not stay within the liquid environment [7]. The flagellum contributes to bacterial pathogenicity by touching surfaces to adhere to either normal or degenerating tissue cells and promoting the biofilm formation to help the bacterial colonize to host [13,14,15]. Understanding the adaptation of swarming motility is essential for understanding the spread and pathogenicity of *V. parahaemolyticus* in the environment.

Motility has been identified as an essential virulence for the survival and colonization of *V. parhaemolyticus* [12,16]. The expression of flagellar genes is tightly regulated by the transcription regulators and sigma factors. The expression of polar flagellar genes is continuous, whereas the lateral flagellar genes are induced by surface growth, iron limitation, calcium presence, and inhibition of polar flagellum [17,18,19]. *CalR,* which is restrained by calcium acting as a LysR-type transcription factor in *V. parahaemolyticus*, negatively regulates *laf* genes, resulting in inhibiting the swarming motility [19]. The T3SS1 AraC-type regulator ExsA can inhibit LafK, the master regulator of the lateral flagellar system in the negative-feedback loop regulated by *calR* in high calcium environments [19]. The quorum sensing master regulator OpaR can inhibit the expression of *laf* gene to controlling the swarming motility [20]. Besides, the transcription factors VPA1701, SwrZ/SwrT, and the two-component system histidine kinase PhoR can down-regulate the expression of lateral flagellar genes for swarming motility in *V. parahaemolyticus* [20,21,22].

Two hundred and thirty genes have been identified that contribute to the colonization of *V. parahaemolyticus* to the small intestine by Tn-seq [23]. Among these 230 genes, 22 transcriptional factor genes have been selected to construct the deletion mutant strains, and investigate their impact to the swimming and swarming motility [21]. We found that VPA0041 was involved in the regulation of swarming motility. Thus, we further investigated the molecular mechanism of VPA0041 in regulating the swarming motility of *V. parahaemolyticus*. RNA-seq was used to identify the regulon of VPA0041 protein, and the lateral flagellar system was down-regulated in the ∆*vpa0041* mutant strains, which was confirmed by qRT-PCR. Our findings revealed the regulation mechanism of VPA0041, which could determine the swarming motility in *V. parahaemolyticus*.

## 2. Results

### 2.1. VPA0041 Regulated the Swarming Motility of V. parahaemolyticus

**Figure 1:**
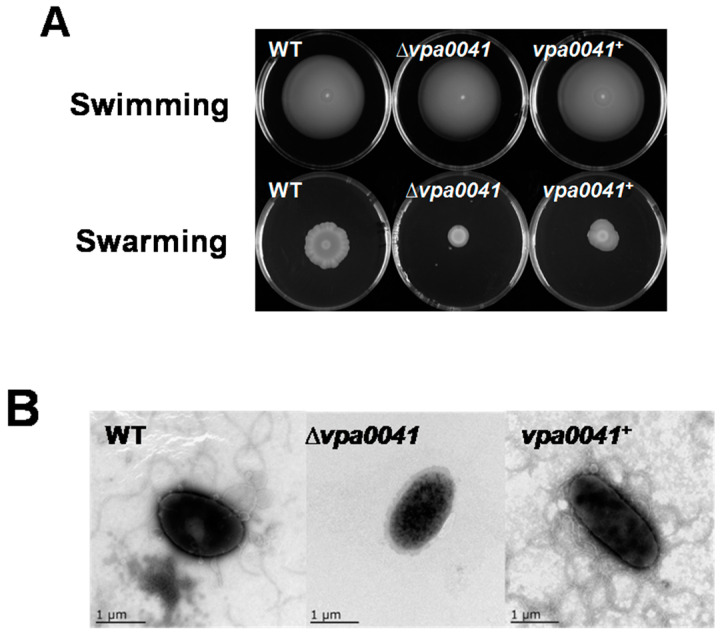
VPA0041 regulates the swarming motility of *V. parahaemolyticus*. (**A**) The *Vibrio parahaemolyticus* WT, ∆*vpa0041* and *vpa0041*^+^ were incubated in swimming plates (LB + 0.3% Agar) at 37 °C and swarming plates (BHI + 1.5% Agar) at 30 °C. (**B**) TEM micrographs show the generation of lateral flagellum in *V. parahaemolyticus* WT, ∆*vpa0041,* and *vpa0041*^+^.

In this study, we investigated the regulation of swarming motility by VPA0041 in *V. parahaemolyticus*. The *vpa0041* deletion mutant strain did not show a significant difference in swimming motility, while the swarming motility was significantly decreased compared to WT on the BHI plated with 1.5% agar (Figure 1A). The complemented strains *vpa0041*^+^ restored the swarming ability, which confirmed the role of VPA0041 in swarming motility in *V. parahaemolyticus* (Figure 1A). The TEM was used to observe the flagellum of *V. parahaemolyticus* strains, and no flagellum was observed in the Δ*vpa0041* strain, whereas several typical lateral flagellums were observed surrounding WT and *vpa0041*^+^ cells (Figure 1B). These results indicated that the deletion of *VPA0041* could down-regulate the lateral flagellum assembly and the ability of swarming motility.

### 2.2. The Annotation of VPA0041 Protein in V. parahaemolyticus

**Figure 2:**
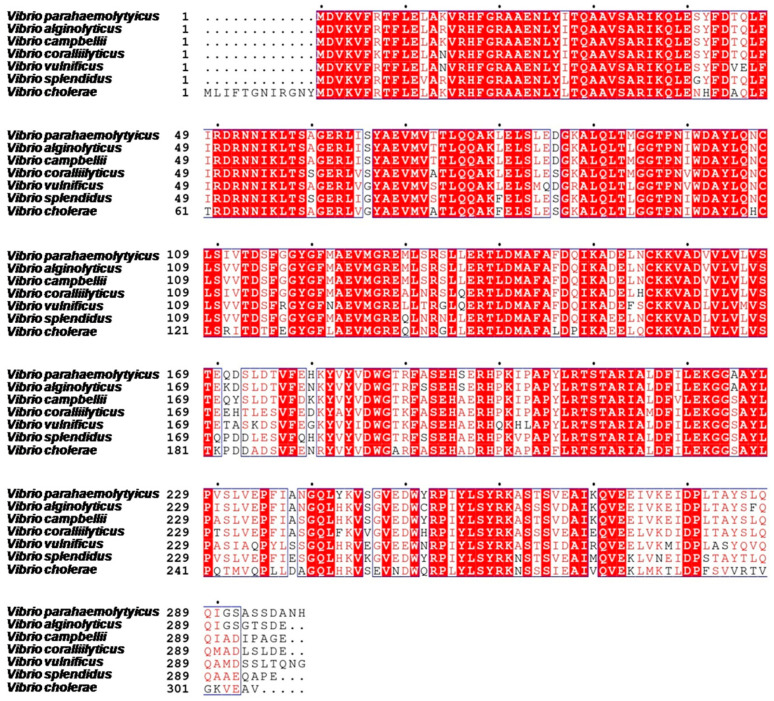
Multiple amino acid sequence alignments of VPA0041 proteins in *Vibrios*. Red rectangular frames indicate the homology of VPA0041 protein between *V. parahaemolyticus* and other Vibrios. The graphs show differences, and dots indicate missing residues.

The study showed that VPA0041 was recognized as a new transcription regulator of the swarming motility in *V. parahaemolyticus*. The *vpa0041* gene was 897 bp in length encoding 299 amino acids belonging to the LysR family, which contained a helix-turn-helix (HTH) domain at the N-terminal and a co-factor binding domain at C-terminal. BlastP results from GenBank indicated that *V. parahaemolyticus* VPA0041 shared 95%, 96%, 87%, 78%, 87%, and 77% identities to N646-3496 in *Vibrio alginolyticus*, VIBHAR_06999 in *Vibrio campbellii*, VIC_002269 in *Vibrio coralliilyticus*, AOT11_01700 in *Vibrio vulnificus*, VISP3789_21993 in *Vibrio splendidus,* and VCA1020 in *Vibrio cholerae,* respectively (Figure 2). This result indicated that the VPA0041 could be highly conserved with primarily the DNA binding domain among the *Vibrio* species.

### 2.3. The Global Transcriptional Analysis of VPA0041 in V. parahaemolyticus

**Figure 3:**
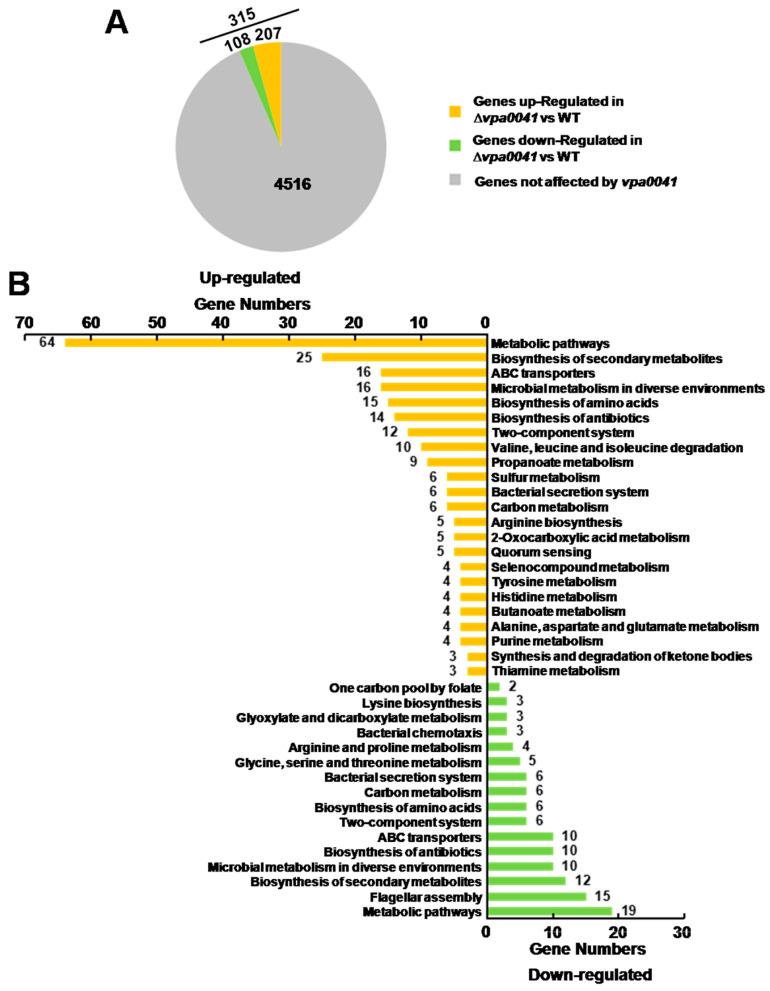
RNA-seq reveals the genes affected by *vpa0041*. (**A**) The pie chart shows the number of genes affected by *vpa0041* according to ∆*vpa0041* compared to WT cultured in BHI agar plates. (**B**) KEGG analysis reveals the different pathways associated with *vpa0041* in ∆*vpa0041* compared to WT. The orange shows the up-regulated genes; the green shows the down-regulated genes.

VPA0041 protein was identified as a transcription factor engaged in regulating the swarming motility in *V. parahaemolyticus*. RNA-seq was used to identify the regulon of VPA0041 and further elucidate the potential mechanisms of VPA0041-regulated swarming motility. Comparison of the RNA-seq data for the WT and Δ*vpa0041* incubated on the BHI agar plates showed that 315 genes were significantly regulated by VPA0041 (log_2_FC ≤ −1 or log_2_FC ≥ 1; *p* < 0.05), 207 genes were up-regulated and 108 genes down-regulated in Δ*vpa0041* compared to WT (Figure 3A).

Kyoto Encyclopedia of Genes and Genomes (KEGG) pathway analysis was performed to identify the specific pathways regulated by VPA0041. Upwards of 100 pathways were activated, and several pathways are shown in Figure 3B. A total of 18 genes were assigned to the two-component system KEGG pathway (12 genes up-regulated and 6 genes down-regulated). Twelve genes associated with the bacterial secretion system were regulated by VPA0041 (six genes up-regulated and six genes down-regulated), including the type III secretion system 1 and type VI secretion system 2. The metabolic pathway (64 genes up-regulated and 19 genes down-regulated) and the ABC transporters pathway (16 genes up-regulated and 10 genes down-regulated) are also identified in the regulon of VPA0041. Notably, 15 genes of the lateral flagellar system and three genes of bacterial chemotaxis were down-regulated in the Δ*vpa0041* compared with the WT, indicating that VPA0041 protein might act as a positive regulator for the swarming motility in *V. parahaemolyticus*. The transcript levels of polar flagellar genes were not influenced by VPA0041 protein. These results demonstrated that VPA0041 maybe play an essential role in the multiple metabolic and virulence pathways in *V. parahaemolyticus*.

### 2.4. VPA0041 Regulates the Expression of the Lateral Flagellar Genes

**Figure 4:**
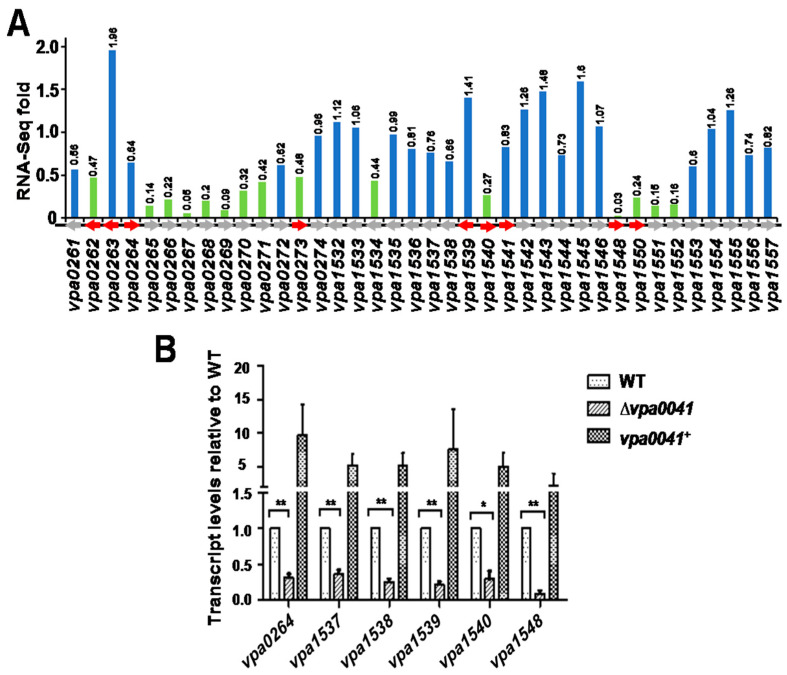
VPA0041 regulates the expression of lateral flagellar genes. (**A**) RNA-seq performed the expression pattern of all lateral flagellar genes. The green columns indicate the significant down-regulated genes in Δ*vpa0041* compared to WT strain (fold change (Δ*vpa0041*/WT) ≤ 0.5 and *p* < 0.05), the blue columns indicate the gene not influenced by VPA0041. The gray arrows indicate the configuration of the lateral flagellar genes on the chromosome, and the red arrows indicate the predicted promoters. (**B**) The expression pattern of several lateral flagellar genes in WT, ∆*vpa0041,* and *vpa0041*^+^ was examined by qRT-PCR. Data showed the mean ± SE from three independent experiments. ** *p* < 0.01; * *p* < 0.05, calculated by Student’s *t*-test.

RNA-seq analysis indicated that the lateral flagellar genes were down-regulated in the regulon of VPA0041. Among the 38 lateral flagellar system genes, 15 genes were significantly decreased in the Δ*vpa0041* compared to the WT (Figure 4A). Furthermore, qRT-PCR showed that the expression of *vpa0264*, *vpa1537*, *vpa1538*, *vpa1539*, *vpa1540,* and *vpa1548* were significantly down-regulated in Δ*vpa0041* compared with the WT; and the complemented strains *vpa0041*^+^ restored the transcript level of these genes to the WT (Figure 4B). These results indicated that VPA0041 could regulate the expression of lateral flagellar genes to mediate the swarming motility in *V. parahaemolyticus*.

### 2.5. VPA0041 Directly Bound to the Promoters of vpa0264, vpa1548, and vpa1550 to Activate the Lateral Flagellar System

**Figure 5:**
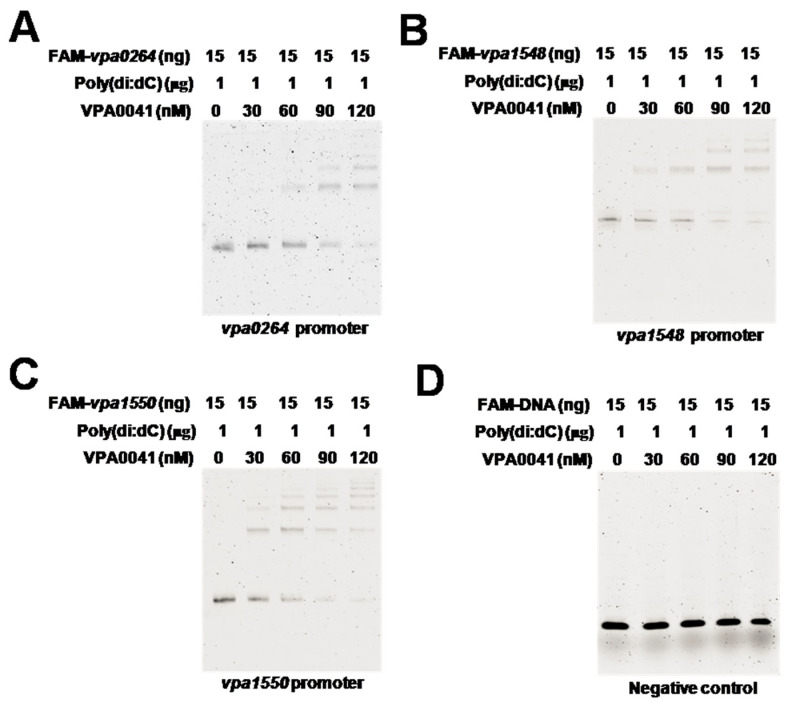
VPA0041 bound widely to the lateral flagellar promoters. (**A**–**D**) EMSA was performed with the DNA fragment from the three lateral flagellar promoters: *vpa0264*, *vpa1548,* and *vpa1550*. The increasing amounts of VPA0041 protein were used as indicated, and 15 ng of each probe with 1 μg non-specific competitor DNA poly(dI:dC). The *gyrB* gene was used as a negative control.

Thirty-eight lateral flagellar system genes were divided into 2 clusters located in chromosome II of *V. parahaemolyticus* [21]. Bioinformation analysis showed that the lateral flagellar system might contain nine promoters, shown in Figure 4A. The *vpa0264-vpa0274* cluster, *vpa1548* gene, and *vpa1550-vpa1557* cluster were significantly down-regulated in the Δ*vpa0041* strain. EMSA results showed that the VPA0041 protein was directly bound to the promoter regions of *vpa0264*, *vpa1548,* and *vpa1550* in a concentration-dependent manner (Figure 5A–C). The negative probe remained unbound with the highest concentration of VPA0041 protein (Figure 5D).

**Figure 6:**
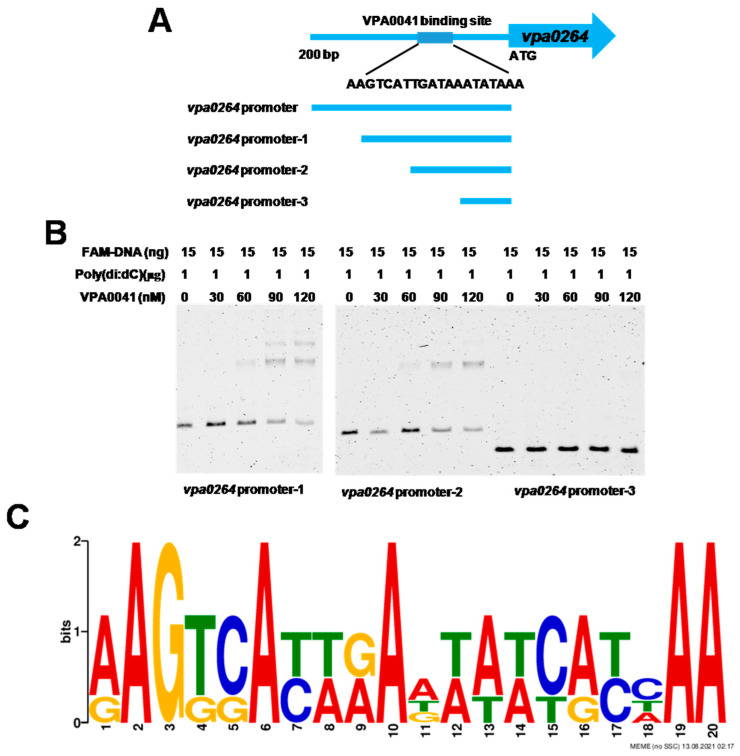
Identification of VPA0041-binding sites in the promoter of *vpa0264*. (**A**) The promoter region of *vpa0264*. Four DNA probes of the *vpa0264* promoters were designed to analysis the binding sited of VPA0041. (**B**) EMSA analysis of VPA0041 binding sites with the various truncations. (**C**) The binding motif of VPA0041 protein generated by the MEME Suit tool.

The promoter region of *vpa0264* was shown in the Figure 6A. Four different probes were used to analyze binding sites of VPA0041 in the promoter of *vpa0264*. The EMSA results indicated that VPA0041 could bind to the *vpa0264* promoter, promoter-1 and promoter-2 but not promoter-3 (Figure 5A and Figure 6B). Then the MEME-Suit tool (http://meme-suite.org; accessed on 13 August 2021) was used to identify the binding motif of the VPA0041 protein based on the sequences of *vpa0264*, *vpa1548,* and *vpa1550* promoters. As showed in the Figure 6C, the specific binding motif of the VPA0041 is a TA-rich region, and the VPA0041-binding site in the *vpa0264* promoter was 5′-AAGTCATTGATAAATATAAA-3′ (Figure 6A). In addition, the predicted VPA0041-binding sites in the promoters of *vpa1548* and *vpa1550* were showed in the Figure S1A. Then we also used the EMSA to verify the binding site of VPA0041 in the promoter of *vpa1548* and *vpa1550*. However, the VPA0041 protein still could bind to all of the different probes of *vpa1548* or *vpa1550* promoter (Figure S1B–E). Our current results indicated that VPA0041 protein could directly bind to the promoters of *vpa0264*, *vpa1548,* and *vpa1550* to induce the expression of the lateral flagellar system genes and responsible for the swarming motility of *V. parahaemolyticus*.

## 3. Discussion

The expression of lateral flagellar genes has three tiers (classes I, II, and III) and is tightly regulated by LafK dependent on σ^54^ and σ^28^ in *Vibrio parahaemolyticus* and other Vibrios [19,24]. Apart from the regulation of LafK to the lateral flagellar genes, the other transcriptional factors independent of σ^54^ and σ^28^ have also been identified to control the swarming motility behavior of *Vibrio parahaemolyticus*, these transcriptional factors including VPA1701, SwrT/SwrZ, OpaR, ScrG/ScrC, ToxR, OxyR, CalR, and HU [19,20,21,25,26,27,28,29]. In this study, we identified a novel transcriptional factor, VPA0041, which regulated the swarming motility, and 15 flagellar assembly genes were down-regulated by VPA0041. EMSA confirmed that the VPA0041 protein could directly bind to the promoters region of *vpa0264-vpa0272* cluster, *vpa1548* gene, and *vpa1550-vpa1557* cluster (Figure 5). The *vpa0264-vpa0272* cluster could encode the FlgBCDEFGHIJ protein, which could form the rod, P/L ring, and hook of the flagellum [9]. The *vpa1548* gene could encode the FliC, which could form the filament of the flagellum [30]. The *vpa1550* and *vpa1551* genes could encode the FliDS protein, forming the filament cap of the flagellum [9,30]. Notably, the swimming motility was not affected in Δ*vpa0041*, but no polar flagellum was observed in Δ*vpa0041*. This result was due to that the bacterial were cultured in BHI agar and collected to observe the lateral flagellum, while the polar flagellum could not be induced in this condition. These results demonstrated that the VPA0041 could down-regulated the expression of *flgBCDEFGHIJ*, *fliC*, and *fliDS* to block the generation of the lateral flagellum.

VPA0041 is a transcription factor belonging to the LysR family, which is an abundant prokaryote and contains a conserved HTH motif at the N-terminal and a co-factor binding domain at the C-terminal [31]. LysR-type transcriptional regulators are distributed among diverse bacteria, regulating various biological processes, such as virulence, metabolism, quorum sensing, and motility [32,33,34]. In this study, we identified a novel LysR family transcription factor, VPA0041, capable of regulating the swarming motility of *V. parahaemolyticus* (Figure 1). RNA-seq results indicated that the VPA0041 protein could regulate the expression of lateral flagellar system genes, and confirmed by qRT-PCR (Figure 4). Moreover, the expression levels of lateral flagellar genes in the complementary strain were higher than those of the WT (Figure 4B), but the swarming motility of the complementary strain was weaker than that of WT (Figure 1A). This result may be due to that the high expression of VPA0041 protein increased the transcript levels of the lateral flagellar genes in complementary strain. However, the mRNA of the lateral flagellar genes translated to protein may be limited in the bacterial and fewer proteins were expressed, leading to the swarming motility of complementary strain was weaker than that in WT. In addition, the VPA0041 protein could directly bind to the promoter region of the lateral flagellar system to regulate the expression of the lateral flagellar genes and mediate the swarming motility in *V. parahaemolyticus* (Figure 5).

Results showed that the LysR family transcription factor VPA0041 could down-regulate the swarming motility in *V. parahaemolyticus*. Previous studies showed that LysR-type protein could act as a transcription regulator regulating the expression of motility in various bacteria. In *E. coli*, the LysR-type transcription regulators LrhA and HdfR could actively regulate the expression of FlhDC, which was the master regulator of the flagellar system and responsible for motility [35,36]. RovM was identified to be homologous to LrhA and regulated the cell invasion, virulence, and motility in *Yersinia pseudotuberculosis* [37]. Conversely, HexA was identified as a LysR-type transcription factor, which could inhibit the expression of *fliA* and *fliC*, responsible for motility ability [38]. The LysR-type transcription factor activates or inhibits the expression of the flagellar system genes. In our study, the LysR family protein VPA0041 could directly bind the promoter region of *vpa0264*, *vpa1548,* and *vpa1550*; these genes were involved in the lateral flagellar system in *V. parahaemolyticus*. Besides, our results identified the specific binding site of VPA0041 protein was 5′-AAGTCATTGATAAATATAAA-3′ in the promoter *vpa0264*, which was a TA-rich region (Figure 6). The EMSA results shown that VPA0041 protein could bind to all DNA probes of *vpa1548* or *vpa1550* promoter (Figure S1D,E), indicating that more than one binding sites were existence in these promoters. There were multiple bands in the *vpa1550* promoter-1 to -3, while only one band was found in the *vpa1550* promoter-4 (Figure S1E). These results indicated that one binding site might located in the promoter-4 region which was consistent with binding site predicted by MEME, and another binding site may existence in the region of 50–100 bp relative to ATG. Another LysR family protein GcdR also shown multiple bands and identified two specific binding sites in the promoter, one of them was a TA-rich region [39].

Concerning the pathogenesis of *V. parahaemolyticus*, a wide variety of virulence factors, including thermostable direct hemolysin (TDH), TDH related hemolysin (TRH), adhesins, motility, biofilm, Type III secretion system, and Type VI secretion system, have been comprehensively studied [6,40,41,42,43]. Previously studies have demonstrated the relationship between the motility and pathogenicity in several bacterial species including *V. parahaemolyticus*, *Vibrio alginolyticus*, *Acinetobacter baumannii*, and Lysobacter [21,44,45,46,47,48,49]. Interestingly, our RNA-seq results showed that VPA0041 significantly regulated 315 genes on the BHI agar plate (Figure 3). The KEGG pathway analysis also indicated that several virulence-associated pathways were enhanced, including the T3SS1, T6SS1, and lateral flagellar system. Previous studies reported that the lateral flagellum and killing function of T6SS was induced on the agar plate [42,50], which was consistent with our RNA-seq results. T3SS1 is related to motility, biofilm formation, cytotoxicity and contributes to the survival of *V. parahaemolyticus* in the environment [51,52]. Additionally, these virulence-associated genes were also regulated by VPA0041, indicating that the VPA0041 might be a virulence regulator in *V. parahaemolyticus* (Figure 3). The molecular regulation mechanisms of VPA0041 to T3SS1 and T6SS1 need to be further elucidated.

In summary, this study demonstrated that VPA0041 was required for swarming motility and might be beneficial for the survival and pathogenesis of *V. parahaemolyticus* in the surface or tissue environment. This study provides evidence of the participation of the VPA0041 in regulation of the swarming motility of *V. parahaemolyticus*.

## 4. Materials and Methods

### 4.1. Bacterial Strains, Plasmids, and Growth Conditions

All bacterial strains and plasmids were listed in Table 1, and the primers used in this study were listed in Table 2. *V. parahaemolyticus* RIMD2210633 WT and its derivatives, as well as *E. coli,* were incubated in Luria-Bertani (LB) at 37 °C while shaking at 220 rpm. When necessary, the carbenicillin (Carb, 50 μg/mL), chloramphenicol (Cm, 25 μg/mL), kanamycin (Km, 50 μg/mL), and isopropyl β-D-1-thiogalactopyranoside (IPTG, 1 mM) were added to the medium.

### 4.2. Construction of vpa0041 Mutant and Complemented Strains

The construction of *vpa0041* deletion mutant and complemented strains were completed as previously described [57]. The primers for the construction of the *vpa0041* mutant strain were list in Table 2. The PCR-amplified DNA products of up-stream (627 bp) and down-stream (651 bp) were generating to the overlap fragments (1278 bp) by overlap PCR. The overlapped DNA products were inserted into the pDM4 plasmid and transformed into *E. coli* SM10 *λpir*. Subsequently, the recombinant plasmid Δ*vpa0041*::pDM4 was transformed into the WT by conjugation and selected in the LB agar with Carb and Cm. Finally, the second cross-over recombination was selected on the LB agar containing 10% sucrose. The mutant strain (*∆vpa0041*) was verified by PCR (*vpa0041*-out/in-F/R) and sequencing.

The ribosome binding site (RBS) and open reading frame (ORF) regions of the *vpa0041* gene were amplified by PCR with specific primers (*vpa0041*-F/R) and cloned into an expression vector pMMB207. The recombinant plasmid (*vpa0041*::pMMB207) was then transformed into *E. coli* SM10*λpir* and conjugated into the *vpa0041* mutant strain; PCR confirmed this with specific primers (pMMB207-F/R) and sequencing. The expression of VPA0041 was induced by adding 0.1 mM IPTG. 

### 4.3. The Motility Analysis

WT, ∆*vpa0041* and *vpa0041*^+^ were grown in LB medium for 12 h and diluted into new fresh LB medium until OD_600_ of 1.0. Bacterial suspensions measuring 2 μL were dropped onto LB plates with 0.3% agar at 37 °C for 12 h, and Brain Heart Infusion (BHI) plates with 1.5% agar at 30 °C for 24 h to observe the swimming motility and swarming motility, respectively. Three independent experiments were carried out for the motility assay.

### 4.4. The Transmission Electron Microscope Analysis

The WT ∆*vpa0041* and *vpa0041*^+^ strains were incubated on swarming plates for 24 h. The bacteria were then gently washed by 0.01 M PBS into 2 mL Eppendorf tubes. Subsequently, the suspension was added dropwise onto grids for 5 min until the mesh was dry, then the dried samples were covered with 5% uranyl acetate for 30 min in the dry environment before observation by transmission electron microscopy (JEM 2100, Tokyo, Japan).

### 4.5. qRT-PCR Analysis of the Lateral Flagellar Genes

*V. parahaemolyticus* WT ∆*vpa0041* and *vpa0041*^+^ strains were cultured on BHI plates for 18 h, and cells were scraped and harvested from plates, then the total RNA was extracted by the Bacterial Total RNA Extraction Kit (Shangon Biotech, Shanghai, China). The RNA was then treated with RNase free DNase I (Takara, Tsuruga, Japan) to remove gDNA, and the PrimeScript ^TM^ RT reagent Kit (Takara, Tsuruga, Japan) was used to generated cDNA. qRT-PCR was performed with *SYRB* ^®^ Premix Ex Taq ^TM^ (Takara, Tsuruga, Japan) on the ABI StepOnePlus Real-Time PCR System. The *reverses* transcription reaction mix was performed with 20 μL volume including 10 μL SYBR Premix Ex Taq II, 2 μL cDNA, 1 μL each primer, 6 μL ddH_2_O. Six lateral flagellar genes of each sample were checked by 2^−∆∆Ct^, and the *gyrB* gene was used as a control. The primers were listed in Table 2.

### 4.6. RNA-Seq Analysis

*V. parahaemolyticus* WT and Δ*vpa0041* strains were cultured on BHI agar plates, and the total RNA was extracted by the Bacterial Total RNA Extraction Kit (Shangon Biotech, Shanghai, China). RNA samples were digested using DNase I (Promega, Madison, WI, USA) to remove gDNA. Three parallel RNA of each strain were sequenced by the Illumina HiSeq 2000 platform (GENEWIZ, Suzhou, China). The subsequent procedures and statistical analysis were carried out as previously described [22]. In brief, the clean data were aligned to the reference genome of *V. parahaemolyticus* RIMD2210633 using Rockhopper [58,59]. The significant difference expressed genes were defined as the value of log_2_ foldchange ≥ 1 or ≤ −1, and *p* < 0.05. The differentially expressed genes were annotated to the KEGG pathway database [60]. Raw sequencing reads were deposited in the European Nucleotide Archive database under accession number PRJEB39385.

### 4.7. Purification of VPA0041 Protein

The ORF of *vpa0041* gene was amplified with primers *vpa0041*-his-F/R and cloned into the pET30a with BamHI and XhoI. The recombined plasmid *vpa0041*::pET30a was transformed into *E. coli* BL21 (DE3), and verified by PCR with the primers pET30a-F/R. The *vpa0041*::pET30a/BL21 strain was cultured in LB and the expression of VPA0041 protein was induced by IPTG. The bacteria were cultured in 16 °C with 120 rpm for 12 h, before bacterial pellets were collected, washed twice with the binding buffer (0.5 M NaCl, 20 mM Tris-HCl, 5 mM imidazole, pH 7.9) and lysed by sonicate. VPA0041 protein was purified by His Bind Purification Kit (Novagen, Darmstadt, Germany). 12% SDS-PAGE was used to verify the purity of VPA0041 protein (Figure S2).

### 4.8. Electrophoretic Mobility Shift Assay

The EMSA analysis was performed as previously described [61]. Briefly, the promoter regions of the lateral flagellar genes (*vpa0264*, *vpa1548*, *vpa1550,* and the negative DNA) were amplified with 5′-FAM primers (Table 2). The reaction mix was implemented in 20 μL containing 10 ng DNA probes, 1 μg poly(dI:dC), 4 μL 5 × binding buffer (10 mM NaCl, 0.1 mM DTT, 0.1 mM EDTA, 10 mM Tris, pH 7.4) and an increasing amount of VPA0041-his protein. After incubation at 25 °C for 30 min, the reaction mixes were separated by 6% native PAGE gels for 2 h at 100 V. The gel was scanned by Typhoon FLA 9500 (GE Healthcare, Uppsala, Sweden). 

## Figures and Tables

**Table 1:** Bacterial strains and plasmids used in this study.

Strain or Plasmid	Relevant Characteristics	Reference
*E. coli*		
DH5α λ*pir*	Host for π requiring plasmids	[53]
SM10 λ*pir*	Host for π requiring plasmids, conjugal donor	[54]
BL21(DE3)	Host strain for protein expression	Novagen
*V. parahaemolyticus*		
RIMD 2210633	Clinical isolate. Carb^r^	[23]
∆*vpa0041*	RIMD 2210633, in-frame deletion in *vpa0041*, Carb^r^	This study
*vpa0041^+^*	∆*vpa0041*, pMMB207 expressing the *vpa0041-his* gene, Carb^r^, Cm^r^	This study
Plasmids		
pDM4	Suicide vector, *pir* dependent, R6K, *SacBR*, Cm^r^	[55]
pMMB207	IncQ lacI^q^ Δ*bla* P_tac-lac_ *lacZa*, Cm^r^	[56]
pET30a	Expressing vector, Km^r^	Novagen
*vpa0041*::pDM4	*vpa0041* up and down sequences clones into pDM4, Cm^r^	This study
*vpa0041*::pMMB207	RBS and *vpa0041*-his sequences clones into pMMB207, Cm^r^	This study
*vpa0041*::pET30a	*vpa0041* ORF clones into pET30a, Km^r^	This study

**Table 2:** Primers used in this study.

Primer Name	Primer Sequence (5′ to 3′)	Target
*vpa0041*-up-F	GATAACAATTTGTGGAATCCCGGGAAGAAGAAATGGGTCAGAAGCGTT	*vpa0041* deletion mutant
*vpa0041*-up-R	CGTCCGATGACTTAACATCCATAAACCAACTCCTG	*vpa0041* deletion mutant
*vpa0041*-down-F	GGATGTTAAGTCATCGGACGCAAACCATTAAC	*vpa0041* deletion mutant
*vpa0041*-down-R	AGTGTATATCAAGCTTATCGATACCCGCACAAAGACAGTGAAGGCAAT	*vpa0041* deletion mutant
*vpa0041*-out-F	TTGTTGCGACCGTTTAGCGTATG	*vpa0041* deletion mutant
*vpa0041*-out-R	CGCACAAAGACAGTGAAGGCAAT	*vpa0041* deletion mutant
*vpa0041*-in-F	TACGCAAGCAGCGGTCAGTG	*vpa0041* deletion mutant
*vpa0041-in-R*	GCTCAGAAGCGAAGCGTGTT	*vpa0041* deletion mutant
*pDM4*-F	GGTGCTCCAGTGGCTTCTGTTTCTA	*vpa0041* deletion mutant
*pDM4-R*	CAGCAACTTAAATAGCCTCTAAT	*vpa0041* deletion mutant
*vpa0041*-F	CCGCGAGCTCTAAGGAGGTAGGATAATAATGGATGTTAAGGTCTTTAGAACAT	*vpa0041^+^*
*vpa0041-R*	GCGTCGACTTAGTGATGATGATGATGATGATGGTTTGCGTCCGATGATGC	*vpa0041^+^*
*pMMB207-F*	CTCCCGTTCTGGATAATGTTTTTTG	*vpa0041^+^*
*pMMB207-R*	TCTTCTCTCATCCGCCAAAACAGCC	*vpa0041^+^*
*vpa0041-his-F*	GGAATTCCATATGATGGATGTTAAGGTCTTTAGAAC	VPA0041 expression
*vpa0041*-his-R	GGAATTCCATATGATGGATGTTAAGGTCTTTAGAAC	VPA0041 expression
*pET30a*-F	TAGTTATTGCTCAGCGGTGGC	VPA0041 expression
*pET30a*-R	ACGATGCGTCCGGCGTAGAG	VPA0041 expression
*gyrb-RT-F*	TTACCGTCATGGTGAGCCTG	qRT-PCR
gyrb-RT-R	CACGCAGACGTTTTGCTAGG	qRT-PCR
*vpa0264*-RT-F	GCAGGTTCAGGCCCAGTATT	qRT-PCR
*vpa0264*-RT-R	TCATGTTGAGAAACGTCAGGCT	qRT-PCR
*vpa1537*-RT-F	CGCTTGAGAAAACGACAGTGG	qRT-PCR
*vpa1537*-RT-R	CCTACTAATGCGGTCTCGGC	qRT-PCR
*vpa1538*-RT-F	CACGTACGCACATATCCGGT	qRT-PCR
*vpa1538*-RT-R	ACGAACACCTTGCTCAACCT	qRT-PCR
*vpa1539*-RT-F	ATTAGTGAGGGTGCGCCTTT	qRT-PCR
*vpa1539*-RT-R	GGTGAAGGGAAGGAATGGCA	qRT-PCR
*vpa1540*-RT-F	CAACGCCAGTTCGTCTTAACG	qRT-PCR
*vpa1540*-RT-R	ACGGCCAGTAAAGAGAGGTTG	qRT-PCR
*vpa1548*-RT-F	GCTGGTGGCCTTATCGAAGA	qRT-PCR
*vpa1548*-RT-R	TACTGCGAAGTCTGCATCCAT	qRT-PCR
FAM-F	FAM-TGCCTGCAGGTCGACGAT	EMSA
*gyrB*-EMSA-F	TGCCTGCAGGTCGACGATTGCCTGCAGGTCGACGATGCGCGCG	EMSA
*gyrB*-EMSA-R	TGCCAGCGCACCGCTGACCGCAG	EMSA
vpa0264-EMSA-1F	TGCCTGCAGGTCGACGATGTTTGTCCTGTCGAAAGAATTC	EMSA
vpa0264-EMSA-1R	CATGCATCTTTCCTTACAGTCGGCT	EMSA
vpa0264-EMSA-2F	TGCCTGCAGGTCGACGATTAGAGTTTTCCCCCTAATTTT	EMSA
vpa0264-EMSA-3F	TGCCTGCAGGTCGACGATTCCACTCTTGTTTGTAAGTCAT	EMSA
vpa0264-EMSA-4F	TGCCTGCAGGTCGACGATGTATCTTGTTTGTATCTTGGCG	EMSA
vpa1548-EMSA-1F	TGCCTGCAGGTCGACGATGCCGATCAAAGCACATCGGAA	EMSA
vpa1548-EMSA-1R	CATCTTAGTCTCCTTAGTTTATCAC	EMSA
vpa1548-EMSA-2F	TGCCTGCAGGTCGACGATCTTAGTGGAATGCAAGTCACT	EMSA
vpa1548-EMSA-3F	TGCCTGCAGGTCGACGATAAATTTTTAATTTTCAAATTA	EMSA
vpa1548-EMSA-4F	TGCCTGCAGGTCGACGATAGTGACTAGGGAATATCCCAAG	EMSA
vpa1550-EMSA-1F	TGCCTGCAGGTCGACGATTTAGTCGCCTTGTCTTATAGGGA	EMSA
vpa1550-EMSA-1R	CACGAGCTTACTCCCTCTCTCATTG	EMSA
vpa1550-EMSA-2F	TGCCTGCAGGTCGACGATGGAACACATAAGGTGGAAAATAC	EMSA
vpa1550-EMSA-3F	TGCCTGCAGGTCGACGATCAAGTCAATGTTTTAAAAGAAT	EMSA
vpa1550-EMSA-4F	TGCCTGCAGGTCGACGATAGACATACTTTCAAGGCATAGAG	EMSA

## Data Availability

Raw sequencing reads of RNA-seq were deposited in the European Nucleotide Archive database under accession number PRJEB39385.

## Supplementary Materials

The following supporting information can be downloaded at: https://www.mdpi.com/article/10.3390/pathogens11040453/s1. Figure S1: Identify the specific binding sites of VPA0041 protein. (A) The promoter regions of the VPA0041 directly regulated genes, *vpa0264*, *vpa1548*, and *vpa1550*. The conserved VPA0041-binding site is indicated by the blue underline. The translation start site is labeled with the red. (B,C) The promoter regions of *vpa1548* (B) and *vpa1550* (C). (D,E) EMSA analysis of VPA0041 binding sites with the various truncations; Figure S2: SDS-PAGE of VPA0041 protein. 12% acrylamide gel was used to verify the purity of VPA0041 protein. M represent for marker, 1 represent for VPA0041 protein.
