# Supplementary material for: A Novel Transcription Factor VPA0041 Was Identified to Regulate the Swarming Motility in Vibrio parahaemolyticus
Source: Pathogens. 2022 Apr 10;11(4):453. doi: 10.3390/pathogens11040453 (PMC9029033; doi:10.3390/pathogens11040453)
Supplement: Supplementary file 1 [file pathogens-11-00453-s001.zip › pathogens-1669212-supplementary.pdf]

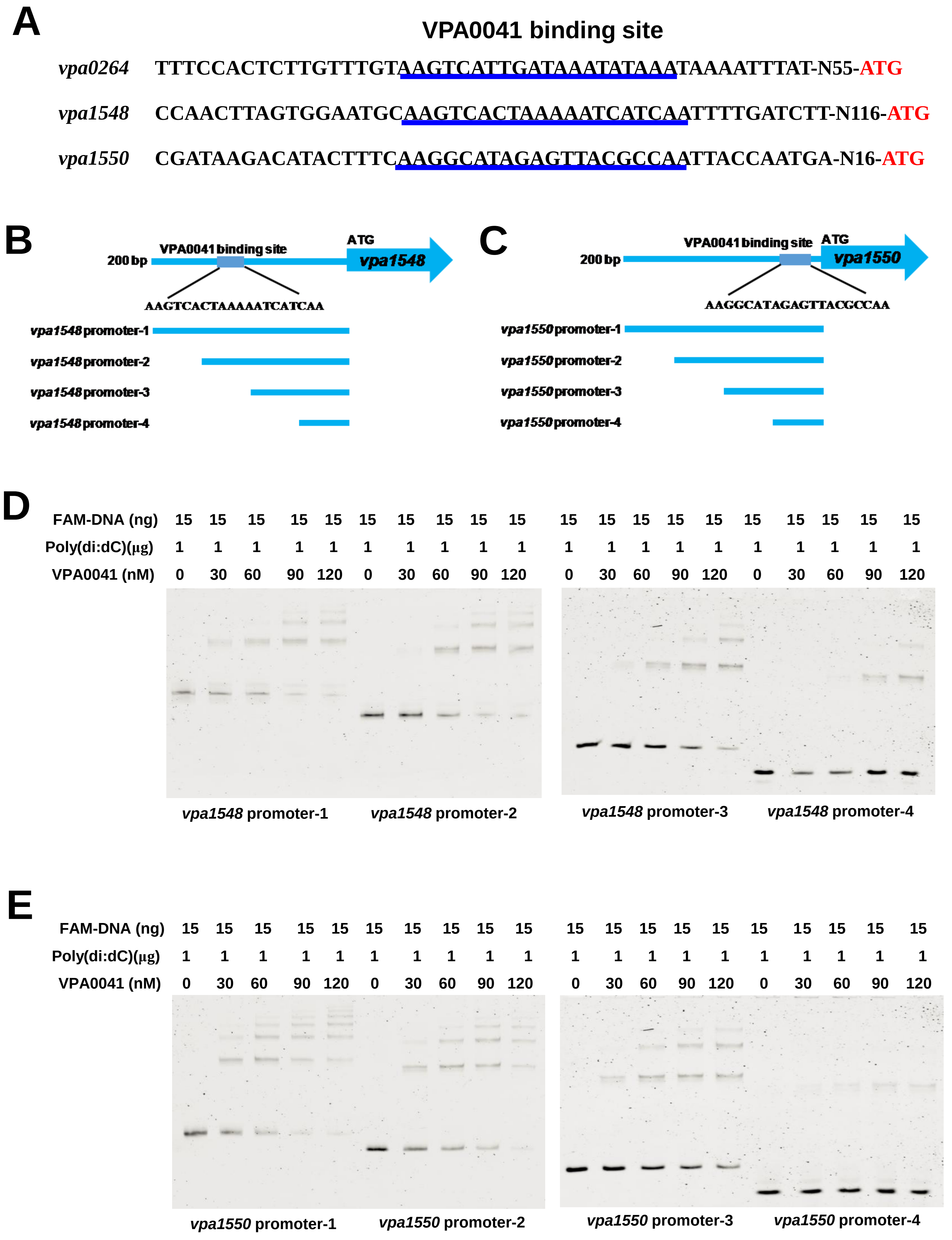
A
VPA0041 binding site
vpa0264 TTTCCACTCTTGTTTGTAAGTCATTGATAAATATAAATAAAATTTAT-N55-ATG
vpa1548 CCAACTTAGTGGAATGCAAGTCACTAAAAATCATCAATTTTGATCTT-N116-ATG
vpa1550 CGATAAGACATACTTTCAAGGCATAGAGTTACGCCAATTACCAATGA-N16-ATG
B
200 bp
VPA0041 binding site
ATG
vpa1548
AAGTCACTAAAAATCATCAA
vpa1548 promoter-1
vpa1548 promoter-2
vpa1548 promoter-3
vpa1548 promoter-4
C
200 bp
VPA0041 binding site
ATG
vpa1550
AAGGCATAGAGTTACGCCAA
vpa1550 promoter-1
vpa1550 promoter-2
vpa1550 promoter-3
vpa1550 promoter-4
D
FAM-DNA (ng) 15 15 15 15 15 15 15 15 15 15 15 15 15 15 15 15 15 15 15 15
Poly(di:dC)(μg) 1 1 1 1 1 1 1 1 1 1 1 1 1 1 1 1 1 1 1 1
VPA0041 (nM) 0 30 60 90 120 0 30 60 90 120 0 30 60 90 120 0 30 60 90 120
vpa1548 promoter-1
vpa1548 promoter-2
vpa1548 promoter-3
vpa1548 promoter-4
E
FAM-DNA (ng) 15 15 15 15 15 15 15 15 15 15 15 15 15 15 15 15 15 15 15 15
Poly(di:dC)(μg) 1 1 1 1 1 1 1 1 1 1 1 1 1 1 1 1 1 1 1 1
VPA0041 (nM) 0 30 60 90 120 0 30 60 90 120 0 30 60 90 120 0 30 60 90 120
vpa1550 promoter-1
vpa1550 promoter-2
vpa1550 promoter-3
vpa1550 promoter-4

Figure S1

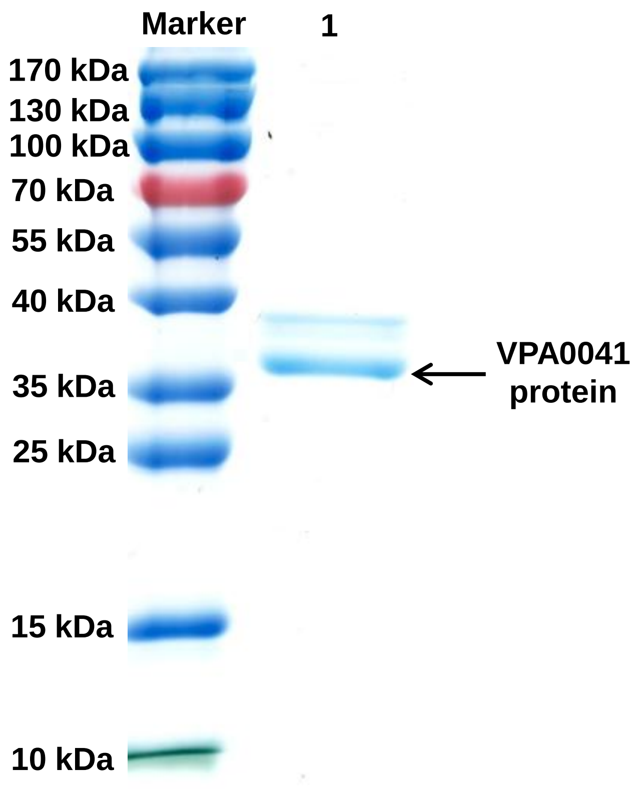
Marker
1
170 kDa
130 kDa
100 kDa
70 kDa
55 kDa
40 kDa
35 kDa
25 kDa
15 kDa
10 kDa
VPA0041 protein

Figure S2
